# Novel Crabtree negative yeast from rumen fluids can improve rumen fermentation and milk quality

**DOI:** 10.1038/s41598-021-85643-2

**Published:** 2021-03-18

**Authors:** Chanon Suntara, Anusorn Cherdthong, Suthipong Uriyapongson, Metha Wanapat, Pin Chanjula

**Affiliations:** 1 Tropical Feed Resources Research and Development Center (TROFREC), Khon Kaen, 40002 Thailand; 2 Department of Animal Science, Faculty of Agriculture, Khon Kaen University, Khon Kaen, 40002 Thailand; 3 Animal Production Innovation and Management Division, Faculty of Natural Resources, Prince of Songkla University, Hat Yai Campus, Songkhla, 90112 Thailand

**Keywords:** Biochemistry, Biological techniques, Microbiology, Zoology, Environmental sciences, Environmental social sciences

## Abstract

Upgrading the nutritive value of rice straw (RS) is necessary to increase its contribution to enhancing meat and milk production. Present work verified whether novel Crabtree negative yeast inoculant could promote RS utilization, rumen fermentation, and milk quality in tropical crossbred lactating Holstein cows. The new stain of Crabtree negative yeasts (*Pichia kudriavzevii* KKU20 and *Candida tropicalis* KKU20) was isolated from the rumen of dairy cattle. This study used 6 multiparous crossbreds between Holstein Frisian × Zebu dairy cows in their mid-lactation period. Dairy cows were randomly allocated to three ensiled RS with various yeast stains including *Saccharomyces cerevisiae*, *P. kudriavzevii* KKU20, and *C. tropicalis* KKU20 according to a 3 × 3 replicated Latin square design. Crabtree-negative yeast (*P. kudriavzevii* and *C. tropicalis*) increased the apparent digestibility of dry matter by about 6.9% when compared with Crabtree-positive yeast (*S. cerevisiae*). Bacterial populations were highest with ensiled RS by *C. tropicalis* KKU20. Ensiled RS with Crabtree-negative yeasts were significantly increased with total volatile fatty acids, but they did not affect volatile fatty acid profiles. Milk protein precentage was highest at 35.6 g/kg when *C. tropicalis* was fed, and lowest when applied with *S. cerevisiae* and *P. kudriavzevii* KKU20 in ensiled RS at 34.5 and 34.1 g/kg, respectively. Thus, feeding ensiled RS with novel Crabtree negative yeast could improve RS digestion, rumen fermentation, and milk protein content in dairy cows.

## Introduction

As a result of rice production, million tons of rice straw (RS) are generated as a by-product; nevertheless, with low nutritional values^1^. Various approaches are applied to enhance the nutritional value and improve its utilization in dairy cow's nutrition^2^. The biological approaches are the most common with more harmless-treated RS^3,4^. For several years, yeast has become an innovative biological model organism for enhancing animal efficiency and is the traditional practice for ruminant feed additives^5^. Yeasts have been especially beneficial for single-cell protein creation and simply acceptable as their biomass has been used by ruminants in the form of fermented feed. In many studies, using yeast fermented with RS has been shown to enhance their nutritional value, silage quality, and nutrient digestibility^6^. *S. cerevisiae*, rapidly converts molasses and urea to provide biomass and greater nutrients from the whole-cell when added with oxygen (O_2_) during the proliferation process^7^. Wanapat et al.^8^ stated that *S. cerevisiae* significantly increases crude protein (CP) in feedstuff via cell proliferation during the fermentation process and it provides essential amino acids, particularly lysine and methionine, for dairy cattle. Previous studies explained the benefit of live yeast in that it could provide a positive effect on feed utilization and performance production in the ruminants^9,10^.

Although *S. cerevisiae* has many benefits, several limitations have been reported, particularly that it produces low cell biomass^6^. Under aerobic conditions, *S. cerevisiae* exhibits alcoholic fermentation more than producing biomass. The “Crabtree-positive yeasts” are those that represent this characteristic^11^. Under excessive glucose and even aerobic conditions, Van Urk et al.^12^ revealed that *S. cerevisiae* had a limited proliferation capacity. Similarly, Wardrop et al.^13^ revealed that when cultivated with excessive glucose in a media solution, *S. cerevisiae* provides 7 times lower biomass compared to other strains. This phenomenon restricts the chances of animals to receive highly nutritious from yeast biomasses such as protein, essential amino acids, and vitamins. Consequently, it is important to extend the scope of research, and studying other yeast strains should be further improved. Crabtree-negative yeasts might be the most interesting option, as they have the special characteristic of limited fermentative products, biomass and carbon dioxide are the sole products under excessive glucose and aerobic conditions^11^. Unlike Crabtree positive yeast, excessive glucose did not inhibit affected on ethanol production. The pyruvate has used another channel for converted to cytosolic-acetyl coA via acetaldehyde and acetate through pyruvate dehydrogenase (PDH) bypass channel into mitochondria^14^. Consequently, high yeast biomass production more than Crabtree positive yeast may supply essential rumen fermentation factors and be a greatly nutritious feed supplement for ruminants.

Up to now, there is no research available information about the use of Crabtree negative yeast isolate from the rumen to improve RS. It was hypothesized that the novel Crabtree negative yeast inoculant could promote RS utilization, rumen fermentation, and milk quality in dairy cows. This research aimed to determine the effects of novel Crabtree-negative yeast (*Pichia kudriavzevii* KKU20 and *Candida tropicalis* KKU20) from rumen ensiled with RS and study their effect on feed digestion, ruminal fermentation, milk production, and milk composition of tropical crossbred lactating Holstein cows.

## Results

### Chemical composition of feeds

According to the experiment of De Deken^25^ the *P. kudriavzevii* KKU20 and *C. tropicalis* KKU20 was considered as Crabtree negative yeast, while *S. cerevisiae* as Crabtree positive yeast. In Table 1, the ensiled RS with different yeast species contained crude protein (CP) at 58.9 to 71.2 g/kg dry matter (DM) and 8.4 to 8.5 MJ/kg DM of metabolizable energy (ME), while the concentrate diet contained CP at 178.0 g/kg DM and 12.3 MJ/kg DM of ME. The neutral detergent fiber (NDF) content in ensiled RS with *P. kudriavzevii* KKU20 and *C. tropicalis* KKU20 were lower than in *S. cerevisiae* at 12.7% and 12.1%, respectively (Table 1). Fermentation quality of ensiled RS such as pH was ranged from 4.19 to 4.30, while lactic acid (LA), acetic acid (C_2_), butyric acid (C_4_), and ammonia nitrogen (NH_3_-N) were ranged from 19.8 to 21.9, 5.1 to 5.4, 0.81 to 0.82, and 1.8 to 2.0 g/kg DM, respectively.

**Table 1:** Dietary ingredients and chemical composition of different yeast species in ensiled rice straw and concentrate diet.

Item	Ensiled rice straw, g/kg fresh matter			Concentrate diet, g/kg DM
	*S. cerevisiae*	*P. kudriavzevii* KKU20	*C. tropicalis* KKU20	
**Ingredients**				
Cassava ship	–	–	–	470.1
Corn	–	–	–	77.0
Palm kernel meal	–	–	–	100.0
Rice bran	–	–	–	99.0
Soybean meal	–	–	–	200.0
Molasses	500.0	500.0	500.0	33.0
Premix	–	–	–	0.3
Di-calcium phosphate	–	–	–	0.1
Urea	20.0	20.0	20.0	15.0
Salt	–	–	–	5.5
**Chemical composition, g/kg DM**				
DM, g/kg as fed	279.3	275.7	278.7	903.4
OM	871.4	879.0	874.1	941.3
EE	7.8	8.2	7.4	46.2
CP	60.8	58.9	71.2	178.0
NDF	715.3	624.6	629.3	391.1
ADF	452.2	390.8	396.6	135.1
ADL	64.6	60.6	61.6	30.5
HCEL	263.1	233.9	232.8	256.0
CEL	387.6	330.1	335.0	104.6
**Energy content, MJ/kg DM**				
GE	15.1	15.1	15.0	16.1
ME	8.4	8.4	8.5	12.3
**Fermentation quality**				
pH	4.20	4.30	4.21	–
LA, g/kg DM	19.8	21.9	22.1	–
C_2_, g/kg DM	5.4	5.4	5.1	–
C_4_, g/kg DM	0.82	0.82	0.81	–
NH_3_-N, g/kg DM	2.0	2.0	1.8	–

Premix = Vitamins and minerals; A: 10,000,000 IU; Vitamin E: 70,000 IU; Vitamin D: 1,600,000 IU; Fe: 50 g; Zn: 40 g; Mn: 40 g; Co: 0.1 g; Cu: 10 g; Se: 0.1 g; I: 0.5 g.

*EE* ether extract, *DM* dry matter, *CP* crude protein, *NDF* neutral detergent fiber, *ADF* acid detergent fiber, *ADL* acid detergent lignin, *HCEL* hemicelluose, *CEL* cellulose, *GE* gross energy, *ME* metabolizable energy, *LA* lactic acid, *C*_*2*_ acetic acid, *C*_*4*_ butyric acid, *NH*_*3*_*-N* ammonia-N, Hemicellulose = NDF-ADF Cellulose = ADF-lignin Metabolizable energy calculated according to the equation described by Robinson et al. (2004). Crabtree Negative yeast as *P. kudriavzevii* = *Pichia kudriavzevii* and *Candida tropicalis* = *C. tropicalis* Crabtree Positive yeast as *S. cerevisiae* = *Saccharomyce cerevisiae.*

### Feed intake, nutrient intake, and nutrient apparent digestibility

The impacts of different yeast species ensiled RS on the effectiveness of feed utilization in dairy cattle is illustrated in Table 2. The yeast species did not change the RS intake, concentrate diet, and total intake (P > 0.05). Total intake ranged from 111.7 to 121.1 g/kg BW^0.75^. Organic matter (OM) and CP intake were 8.9 to 9.6 kg/day and 1.3 to 1.4 kg/day, respectively, which was not altered among treatments (P > 0.05). Crabtree-negative yeast (*P. kudriavzevii* KKU20 and *C. tropicalis* KKU20) increased the apparent digestibility of DM by about 6.9% when compared with Crabtree-positive yeast (*S. cerevisiae*). However, the data achieved in this study showed that apparent digestibility of OM (OMD), CP (CPD), NDF (NDFD), and acid detergent fiber (ADFD) were not altered among yeast species and ranged from 762.1 to 791.5, 752.0 to 791.5, 601.5 to 641.3, and 492.8 to 525.4 g/kg, respectively. Furthermore, the total digestible nutrients were the same among yeast species and ranged from 734.8 to 767.7 g/kg (P > 0.05).

**Table 2:** Effect of different yeast species in ensiled rice straw on dry mater intake (DMI), nutrient intake and digestibility in tropical crossbred lactating dairy cows.

Items	*S. cerevisiae*	*P. kudriavzevii* KKU20	*C. tropicalis* KKU20	SEM	P*-value*
**Dry matter intake**					
Rice straw silage					
kg/day	3.7	3.8	3.1	0.41	0.17
% BW	1.0	1.0	0.8	0.10	0.11
g/kg BW^0.75^	43.4	45.0	36.5	4.54	0.11
Concentrate diet					
kg/day	5.9	6.3	6.2	0.19	0.11
% BW	1.7	1.8	1.7	0.06	0.27
g/kg BW^0.75^	72.3	76.1	75.2	2.53	0.21
Total intake					
kg/day	9.6	10.1	9.3	0.55	0.25
% BW	2.7	2.8	2.6	0.15	0.27
g/kg BW^0.75^	115.7	121.1	111.7	4.54	0.11
Nutrient intake, kg/day					
OM	9.1	9.6	8.9	0.49	0.24
EE	0.3	0.3	0.3	0.01	0.11
CP	1.3	1.4	1.4	0.05	0.35
NDF	5.1	4.9	4.5	0.32	0.13
ADF	2.5	2.4	2.1	0.18	0.08
Apparent digestibility, g/kg					
DM	701.4^b^	746.5^a^	753.1^a^	15.3	*P* < 0.05
OM	762.1	763.4	791.5	24.4	0.30
CP	752.0	754.8	786.4	29.6	0.34
NDF	601.5	658.8	641.3	28.1	0.17
ADF	492.8	525.4	510.9	21.1	0.34
TDN	734.8	739.0	767.7	23.1	0.23

*EE* ether extract, *DM* dry matter, *CP* crude protein, *NDF* neutral detergent fiber, *ADF* acid detergent fiber, *TDN* total digestible nutrient, *BW* body weight. Crabtree Negative yeast as *P. kudriavzevii* = *Pichia kudriavzevii* and *Candida tropicalis* = *C. tropicalis* Crabtree Positive yeast as *S. cerevisiae* = *Saccharomyce cerevisiae.*

^a,b^Means in the same row with different superscript letters differ (P < 0.01, P < 0.05).

### Effect on rumen pH, NH_3_-N, blood metabolites, and microbial communities

**Figure 1:**
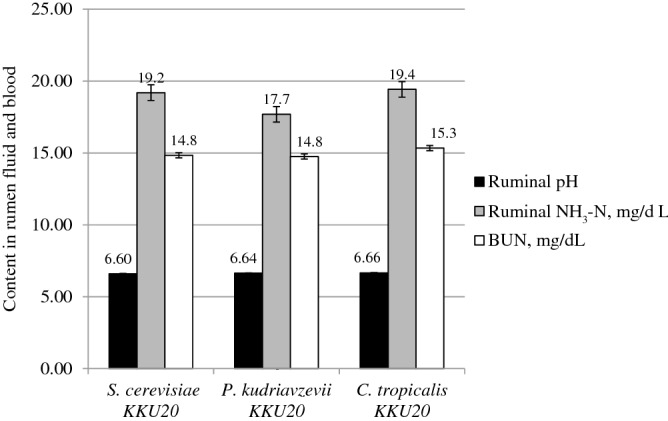
Comparison effects of ruminal Crabtree-Negative yeasts and Crabtree-Positive yeasts on rumen pH, ammonia nitrogen (NH_3_-N) and blood urea nitrogen (BUN) of tropical crossbred lactating Holstein cows.

Table 3 and Fig. 1 illustrate the influence of ensiled RS with various yeast species fed to crossbred lactating dairy cows on ruminal pH, NH_3_-N, blood urea nitrogen (BUN), and microbial communities. Rumen pH was not changed among yeast species, and the pH values were 6.4 to 6.8 (P > 0.05). Ruminal NH_3_-N and BUN ranged from 16.5 to 22.1 mg/dL and 13.3 to 17.3 mg/dL, respectively (P > 0.05). The bacterial populations at both 0 h and 4 h after feeding and the mean value were highest (P < 0.05) with ensiled RS with *C. tropicalis* KKU20 by 9.9, 12.5, and 11.2 Log10 cell/mL, respectively. However, the fungal zoospore and protozoa populations were not affected by any treatments (P > 0.05).

**Table 3:** Effect of different yeast species in rice straw ensiled on ruminal microbial communities in crossbred lactating dairy cows.

Items	*S. cerevisiae*	*P. kudriavzevii* KKU20	*C. tropicalis* KKU20	SEM	*P-value*
**Rumen microbes, cells/mL**					
Bacteria, Log10 cell/mL					
0 h—after feeding	9.2^b^	9.4^ab^	9.9^a^	0.28	*p* < 0.05
4 h—after feeding	11.9^b^	12.2^a^	12.5^a^	0.22	*p* < 0.05
Mean	10.5^b^	10.8^ab^	11.2^a^	0.19	*p* < 0.01
Fungi zoospore, Log10 cell/mL					
0 h—after feeding	7.8	8.1	8.1	0.29	0.31
4 h—after feeding	6.9	6.9	6.9	0.33	0.43
Mean	5.9	5.7	5.6	0.24	0.27
Protozoa, Log10 cell/mL					
0 h—after feeding	4.3	4.6	4.6	0.30	0.38
4 h—after feeding	5.9	6.2	6.2	0.27	0.39
Mean	5.1	5.4	5.3	0.17	1.00

^a,b^Means in the same row with different superscript letters differ (P < 0.01, P < 0.05). Crabtree Negative yeast as *P. kudriavzevii* = *Pichia kudriavzevii* and *Candida tropicalis* = *C. tropicalis* Crabtree Positive yeast as *S. cerevisiae* = *Saccharomyce cerevisiae*.

### Effect on ruminal volatile fatty acid

**Figure 2:**
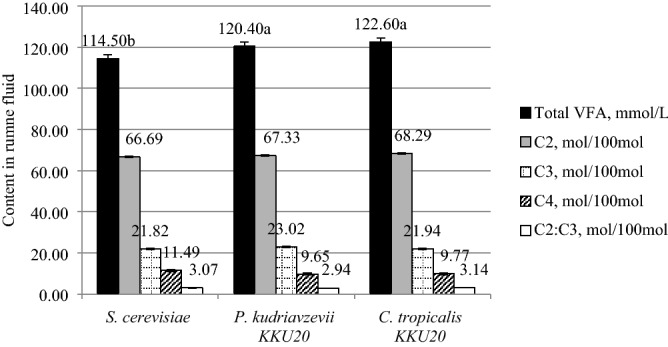
Comparison effects of ruminal Crabtree-Negative yeasts and Crabtree-Positive yeasts on the mean values ruminal total volatile fatty acid (TVFA), acetic acid (C2), propionic acid (C3), butyric acid (C4) and C2:C3 of tropical crossbred lactating Holstein cows.

The TVFA, C_2_, C_3_, C_4_ proportions, and C_2_:C_3_ ratio are illustrated in Table 4 and Fig. 2. Ensiled RS with *P. kudriavzevii* KKU20 and *C. tropicalis* KKU20 were significantly increased with a total VFAs at 0 h (5.15 and 5.06%, respectively), and 4 h (5.07 and 8.83%, respectively) after feeding (P < 0.05) when compared with *S. cerevisiae*, whereas yeasts ensiled RS had no effect on the VFAs’ profile (P > 0.05). The mean value of C_2_, C_3,_ and C_4_ were 67.4, 22.3, and 10.3 mol/100 mol, respectively.

**Table 4:** Effect of different yeast species in rice straw ensiled on concentrations of total volatile fatty acid (VFAs) and their profiles in crossbred lactating dairy cows.

Items	*S. cerevisiae*	*P. kudriavzevii* KKU20	*C. tropicalis* KKU20	SEM	*P-value*
**Total VFA, mmol/L**					
0 h—after feeding	106.7^b^	112.2^a^	112.1^a^	2.38	*p* < 0.05
4 h—after feeding	122.3^b^	128.5^ab^	133.1^a^	4.07	*p* < 0.05
**Volatile fatty acid profiles, mol/100 mol**					
C_2_					
0 h—after feeding	64.9	65.8	65.9	0.86	0.35
4 h—after feeding	68.4	68.8	70.6	1.45	0.20
C_3_					
0 h—after feeding	21.0	21.9	20.7	1.03	0.36
4 h—after feeding	22.6	24.1	23.2	1.13	0.32
C_4_					
0 h—after feeding	14.0	12.2	13.3	1.11	0.20
4 h—after feeding	8.9	7.1	6.2	1.53	0.14
C_2_:C_3_					
0 h—after feeding	3.1	3.0	3.2	0.17	0.48
4 h—after feeding	3.1	2.9	3.1	0.18	0.34

^a,b^Means in the same row with different superscript letters differ (P < 0.01, P < 0.05). *C*_*2*_ Acetic acid, *C*_*3*_ Propionic acid, *C*_*4*_ Butyric acid. Crabtree Negative yeast as *P. kudriavzevii* = *Pichia kudriavzevii* and *Candida tropicalis* = *C. tropicalis* Crabtree Positive yeast as *S. cerevisiae* = *Saccharomyce cerevisiae*.

### Effect on milk production, milk composition, and feed efficiency

**Figure 3:**
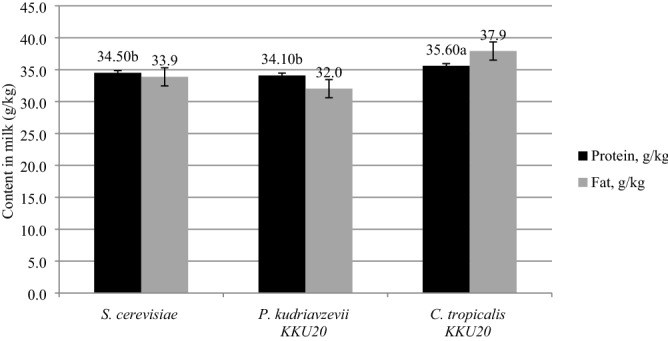
Comparison effects of ruminal Crabtree-Negative yeasts and Crabtree-Positive yeasts on milk protein and milk fat of tropical crossbred lactating Holstein cows.

The effects of ensiled RS with various yeast species on milk production, composition of milk, and feed efficiency in dairy cows are shown in Table 5 and Fig. 3. The yeast strains’ effects were not observed (P > 0.05) on actual milk yields (8.5 to 8.8 kg/h/day), 4.0% fat corrected milk (FCM) (7.6 to 8.3 kg/h/day), and energy corrected milk (ECM) (7.7 to 8.3 kg/h/day). The treatments did not alter the milk composition (P > 0.05); except for when the protein in the milk was highest in the *C. tropicalis* KKU20 fed group at 35.6 g/kg (P < 0.01). Feed efficiency did not changed for any diets (P > 0.05).

**Table 5:** Effect of different yeast species in rice straw ensiled on milk yield, milk composition, feed efficiency and economic efficiency in crossbred lactating dairy cows.

Items	*S. cerevisiae*	*P. kudriavzevii* KKU20	*C. tropicalis* KKU20	SEM	*P-value*
Actual milk yield, kg/h/day	8.5	8.8	8.6	0.47	0.09
4.0% FCM, kg/h/day	7.8	7.6	8.3	0.39	0.11
ECM, kg/h/day	7.8	7.7	8.3	0.30	0.07
Total solids, g/kg	122.7	121.2	126.9	2.91	0.09
SCC, × 10^5^	5.3	4.0	6.6	1.25	0.10
MUN, mg/dL	12.9	13.1	14.7	0.85	0.06
**Feed efficiency**					
Milk/DMI	0.90	0.87	0.92	0.05	0.62
ECM/DMI	0.82	0.76	0.89	0.06	0.07
DCP/Milk	93.4	91.1	97.3	4.02	0.22

*SCC* somatic cell count, *MUN* milk urea nitrogen, *DMI* dry matter intake. *DCP* digestible crude protein.

^a,b^Means in the same row with different superscript letters differ (P < 0.01, P < 0.05). Crabtree Negative yeast as *P. kudriavzevii* = *Pichia kudriavzevii* and *Candida tropicalis* = *C. tropicalis* Crabtree Positive yeast as *S. cerevisiae* = *Saccharomyce cerevisiae*. *FCM* fat corrected milk = 0.432 (kg of milk/day) + 16.23 (kg of fat), *ECM* energy-corrected milk = 7.20 × protein (kg/day) + 12.95 × fat (kg/day) + 0.327 × milk (kg/day).

## Discussion

From NRC^15^, CP and ME requirements of our animal (BW 364 kg, milk yield 8.6 kg/day) increased by about 1,190 g/day and 60.9 MJ/day, respectively. The nutrient composition in feed, especially CP (provide 1,300–1,400 g CP/day) and ME (provide 79.05–84.14 MJ/day) values, were sufficient in our study for supporting dairy cows’ performance. Our study demonstrated that two yeasts were isolated from rumen fluids: *P. kudriavzevii*-KKU20 and *C. tropicalis*-KKU20. The name KKU refers to Khon Kaen University, where the strain was originally isolated, and the number “20” means the year of discovery, 2020. Presently, there are not many experiments has explored the impact of isolated yeasts on the mechanism of fermentation in the rumen. Intanoo et al.^16^ were isolated yeast from rumen fluids of three non-fistulated Thai-Holstein Friesian dairy cows and identified as *Kluyveromyces marxianus* and *Pichia kudriavzevii* for detoxifying aflatoxin B1 (AFB1) and it can apply further to use in animal feed. Correspondingly, Sirisan et al.^17^ found that there are three effective yeasts that could isolate from rumen fluids of Thai-Holstein Friesian dairy cattle including *Pichia kudriavzevii*, *Candida rugosa*, and *Kodamaea ohmeri* which are used as preventing acidosis in dairy cattle diets. Although, these yeast species had previously been isolated and used for the ruminant animal^18^. However, the qualities of these strains on ensiled RS and animal production have not been studied. This is the first time that yeast has been used in animal feed for performance testing.

Ensiled RS with *P. kudriavzevii* KKU20 and *C. tropicalis* KKU20 (Crabtree-negative yeast) was established as having a low fiber content when compared with adding *S. cerevisiae* (Crabtree-positive yeast). The low fiber content can be clarified by the yeast’s ability to release cellulase enzymes and digest fiber during the fermentation process. Suntara et al.^6^ confirmed that *C. tropicalis* KKU20 and *P. kudriavzevii* KKU20 were more capable of releasing cellulase enzymes than *S. cerevisiae* by about 0.7 to 6.8 times, respectively. The experiment on in vitro gas production of ensiled RS at 14 days with the *P. kudriavzevii* KKU20 could decrease the NDF content by about 6.7% when compared with *S. cerevisiae*^6^. Ilmén et al.^19^ discovered yeast isolated from a plant named *C. konsanensis* species could excrete cellulase enzymes and digests fiber, and it is a new yeast strain that had not been reported previously. Similar to our study, *C. tropicalis* KKU20 and *P. kudriavzevii* KKU20 are great potential yeasts to improve feedstuffs and this study is the first report in ruminant nutrition feed research.

The fermentation quality of ensiled RS with different yeast species indicated that the silage was well preserved. The ensiled RS still maintained appropriate pH, high lactic acid content, and a low NH_3_-N level. Acceptable silage was defined by the pH value and the composition of their fermentation products^20^. The pH is the main indicator for evaluating silage quality and our study showed ensiled RS still has a satisfactory score of about 4.1 to 4.3^21^. The pH is highly related to LA content, which in this study showed a consistent range of about 19.8 – 22.1 g/kg DM. LA content in silage should range between 21 to 25 g/kg DM to be considered of high quality, according to Flieg’s score^22^; This is close to the high quality of silage. Our result showed LA content similar to an earlier study by Suntara et al.^6^ who revealed that about 20.53 to 26.14 g/kg DM of LA was produced when ensiled RS with *C. tropicalis* KKU20 and *P. kudriavzevii* KKU20 at 14 days. NH_3_-N concentration in ensiled RS within the range of 1.80 to 2.00 g/kg DM indicated the normal standards for estimating silage. These results are similar to those of Li et al.^23^, who collected information on various types of RS parameters and concluded that RS silage has a NH_3_-N concentration of approximately 1.61 to 2.36 g/kg DM. Other parameters such as C_2_ show great value for preserved silage within the range of 20 to 25 g/kg DM^21^. After the fermentation process, the moisture content should range from 650 to 750 g/kg to be optimum^24^, which in our study showed an average of 722.1 g/kg. Hence, our study proposes that the nutrients in ensiled RS are still well preserved.

The Crabtree effect can be measure from ratio, fermented glucose/ respiration glucose and positive if this ratio is > 1 and negative if the ratio is < 1. According to the experiment of De Deken^25^. This experiment indicated that *Candida tropicalis* is a Crabtree negative yeast. Radecka et al.^26^ stated that *Pichia kudriavzevii* shows ability as a crabtree-negative yeast species. Another feature that shows the difference between Crabtree-positive and negative, with oxygen as the final electron acceptor, respiration is possible under aerobic conditions, but Crabtree-positive yeasts (such as *S. cerevisiae*) would inhibit PDH and produce ethanol instead of respiratory complete resulted in low cell growth^27^. In contrast, “Crabtree-negative” mean yeasts neglect fermentative products, and biomass and carbon dioxide are the primary products under aerobic conditions^12^. According to Suntara et al.^6^ reported that the rate of growth was revealed to be lower in *S. cerevisiae* (Crabtree-positive yeast) than in *Candida tropicalis* and *Pichia kudriavzevii* (Crabtree-negative yeast) (P < 0.01). All of the above reasons were ensured that both yeast strains including *Candida tropicalis* KKU20 and *Pichia kudriavzevii* KKU20 used in this experiment were Crabtree negative yeast.

Crabtree-negative or –positive yeast has no effect on the dry matter intake (DMI). Our results showed that the DMI (range from 2.6 to 2.8% BW) was similar to previous experiments, which is that feeding separate ensiled RS with a concentrate diet to dairy cows creates a DMI range from 2.5 to 3.2% BW^28^. Generally, the amount of RS that an animal intakes daily is limited to around 2.0% BW or less BW^2,29^. Because RS is rich in polysaccharides and has a high lignin and silica content, and thus it limits the voluntary intake^30^. However, Aquino et al.^31^ reported that the amount of RS that ruminants can consume can be as high as 1.2% BW, which is similar to our result of 0.8–1.0%BW. The intake of OM, EE, NDF, and ADF was similar to previous studies of lactating crossbred dairy cows^32,33^. The CP intake (CPI) in this study was also similar with Wanapat et al.^2^, which used lactating crossbred dairy cows (50% Holstein Frisian × 50% Thai native cows) and BW around 365.5 kg, and the CPI was about 1.0 to 1.2 kg/day. Typically, the CP found in tropical forage plants is often relatively low^34^. Especially in RS (%CP) when using a roughage source it can have an effect on the animal’s yield adequacy^35^. Our study showed that ensiled RS with yeast could support protein from yeast to low-quality roughage as RS, and the enhanced intake of protein was high enough to meet the requirement of tropical lactation dairy cows.

The DMD was increased when ensiled RS with Crabtree negative yeast was offered to animals. This strain is outstanding in terms of high proliferation ability and its high yield of cellulase enzymes^6^. The improved digestion may be due to the potential of how rumen microflora are promoted for better digestibility. Yeast is an important biological responder in rumen fermentation, live yeast cells improve microorganisms in rumen^36^ and stabilize pH in the rumen^37^. Habeeb^38^ stated that yeast could provide rumen with biological stimulants, which is necessary for microorganisms’ growth in the rumen and yeast contributes to establishing microbiota^39^ and is why the digestibility was apparently enhanced. This is consistent with Wang et al.^9^, who found that Crabtree-negative yeast as *C. tropicalis* could increase digestion in the in vitro technique and that it generated 3.03% more gas production than did *S. cerevisiae*.

Crabtree-negative yeast did not change the apparent OMD, CPD, NDFD, and ADFD. The NDFD and ADFD are similar among Cabtree-negative and positive yeast (601.50 vs 650.05 g/kg DM and 492.8 vs 518.15 g/kg DM, respectively). Noticeable changes occurred after the silage process was complete, but when the animal intakes the feed, its digestion was not altered. The reason for this is still not clear, but it is possible that yeast does not react directly on RS. Rather, digestion in the rumen occurred by the cooperation of microbes’ synergy until the resulting values were not statistically different. This is similar to an experiment by Suntara et al.^6^, who compared the effect of Crabtree-negative and -positive yeast on ensiled RS on the in vitro gas and confirmed that in the rumen, there was no difference among yeast species in NDFD and ADFD (705.2 vs 703.6 and 464.8 vs 464.4 g/kg DM).

Ensiled RS with the *P. kudriavzevii* KKU20 and *C. tropicalis* KKU20 (Crabtree-negative yeast) could increase bacterial populations when compared to *S. cerevisiae* (Crabtree-positive yeast) by about 4.76%. The ruminal bacterial populations depend on sufficient nutrients or stimulants supply^38^. Yeast is a great supply to stimulate bacteria because it is enriched in essential substances^40^. Previous studies have confirmed that yeast could supply essential amino acids, vitamins, and minerals to increase the ruminal bacteria more than without yeast^41^. The key explanation is that under aerobic conditions, Crabtree-negative yeast may proliferate more than Crabtree-positive yeast since the enzyme mechanism functions differently^11,42^. Suntara et al.^6^ found that at 72 h of incubation time, *P. kudriavzevii* KKU20, *C. tropicalis* KKU20, and *S. cerevisiae* had growth by about 10.02, 9.6, and 8.87 Log cells/mL, respectively. The high amount of Crabtree-negative yeast creates a greater supply of essential nutrients to the rumen bacteria^6^, thus the amount of rumen bacteria is increased in response to the Crabtree-negative yeast.

The ensiled RS with Crabtree-negative yeast has more effect on the total VFAs than with Crabtree-positive yeast by about 6.1% at the mean value. The high production of TVFAs in rumen fluids is related to the amount of ruminal bacteria^43^. The great bacterial population could enhance carbohydrate digestion and then the animal obtains the greater VFAs^44^. This is similar to Castillo-González et al.^45^, who stated that the expansion of rumen microorganisms could increase the quantity of rumen VFAs. Certainly, a high bacterial population in our experiment was related to the Crabtree-negative yeast’s effect. Nonetheless, the direct influence of the Crabtree-negative yeast on rumen bacterial populations was unclear and this hypothesis required further research to be conducted. Expanding the Crabtree-negative yeast population (during the fermentation process) may be more effective than expanding that of the Crabtree-positive yeast (*S. cerevisiae*). This suggests that animals have a greater chance of obtaining stimulants for activating rumen bacteria. In agreement with our results, Wang et al.^9^ compared the effect between Crabtree-negative yeast (*C. tropicalis*) and Crabtree-positive yeast (*S. cerevisiae*) for in vitro gas technique and found that the inclusion of 0.25 × 10^7^ of Crabtree-negative yeast could enhance the total VFAs by 7.7%. Suntara et al.^6^ reported that Crabtree-negative yeast (*P. kudriavzevii* KKU20) increased the total VFAs by 2.3% for in vitro gas study more than Crabtree-positive yeast.

The milk yield and milk composition of ensiled RS with Crabtree-negative yeast did not have any impact. Our study showed that the actual milk yields are about 8.5 to 8.8 kg/h/day, which are slightly lower than previous trials using early to mid-lactation cows (12.6 kg/h/day according to Supapong and Cherdthong^46^; 11.1 kg/h/day according to Wanapat et al.^2^. To produce milk, cows must calve and split their lactation cycle into four phases (early, mid, late lactation, and dry period)^47^. The milk yield response was greater in the early lactation, and in the mid-lactation period, the milk yield begins to decline from its peak^48^. The lower actual milk yields in this study may be because dairy cows were in mid to late lactation (DIM 165.5 to 186.5). Our study indicated that daily protein yields in the milk of the *C. tropicalis* KKU20 group were highest at 35.6 g/kg and lowest when applied with *S. cerevisiae* and *P. kudriavzevii* KKU20 in ensiled RS at 34.5 and 34.1 g/kg, respectively. Milk protein is associated with the feed degradation energy supply as VFAs and microbial crude protein (MCP) synthesis^49^. High amounts of microorganisms in rumen could affect the MCP synthesis. This will be the supplied protein and amino acids (AA) in the small intestine and could enhance the milk protein yields^50^. Our result clearly demonstrated that *C. tropicalis* KKU20 was unique in the highest bacterial population (11.2 Log10 cell/mL), which is why the increase in milk protein yields occurred. Furthermore, there were no differences in milk proteins between *S. cerevisiae* and *P. kudriavzevii* KKU20. This suggests that the influence of Crabtree-negative yeast may play different roles in terms of milk quality. This thought is support by Intanoo et al.^16^, who compared different yeast strains that were in the same group of Crabtree-negative, and found that *P. kudriavzevii* KKU20 decreased daily protein yields in milk by 14.9% when compared with *Kluyveromyces marxianus* in crossbred lactating cows. This yeast species could provide high biomass, which possibly supplies more amino acid sources for milk protein synthesis. This is similar to Wardrop et al.^13^ who stated that *K. marxianus* has an outstanding ability to provide high biomass when compared with other strains. The explanation is limited in regard to *P. kudriavzevii*’s impact on daily protein yields in milk. A few studies have focused on applying non-*S. cerevisiae* to dairy cows and further research about the influence of each strain is required.

Based on this study, the addition of a novel *C. tropicalis* KKU20 inoculant could help enhance RS quality and could promote DMD, the ruminal bacterial population, TVFAs, and the milk protein when compared with other groups. The ultimate objective has been achieved and new knowledge would play an important role in facilitating the implementation of rumen isolated yeast as a feed additive. However, there are certain drawbacks associated with the high-producing lactating cows influenced by *C. tropicalis* KKU20 treated RS, which requires further investigation.

## Methods

The animals participating in this study have been certified by the Khon Kaen University Animal Ethics Committee (Record No. IACUC-KKU 38/62), based on the Ethics of Animal Experimentation of the National Research Council of Thailand. Our study confirmed that all methods were performed in accordance with the relevant guidelines and regulations.

### Isolation and identification procedure

Two fistulated–crossbred Holstein Friesian steers, averaging 350 ± 20 kg body weight, were used as rumen inocula donors. The animals were fed this diet to facilitate microorganism adaptation in the rumen, allowing this to occur for 7 days before the rumen fluid was obtained. According to Sirisan^17^, the ruminal fluid was collected only once on day 7 from each fistulated steer via a rumen cannula at 4 h after the morning feed. Rumen fluids from two steers were mixed well before being filtered through cheesecloth folded to form 4 layers, then into a bottle, and were placed immediately into ice bucket (4 °C). They were then transported to the laboratory within 15 min. In the laboratory, for the total plate count, 1 mL of ruminal fluid from each animal was diluted with 0.85% sodium chloride to 1:10, 1:100, and 1:1000. Each ruminal fluid dilution was spread over a yeast–malt extract (YM) agar plate (HiMedia Laboratories Pvt. Ltd, India), which was then incubated at 39 °C for 72 h. The inoculant plate was dissolved in distilled water and sterilized for 15 min at 121 ͦC by autoclaving. The YM agar consisted of malt extract (3 g/L), yeast extract (3 g/L), peptone (5 g/L), agar (20 g/L), and glucose (10 g/L).

According to Suntara et al.^6^ the isolated yeast has high potential to produce biomass and cellulase enzyme was selected to identification. DNA isolation from potential yeast was performed by boiling lysis buffer cells. The divergent D1/D2 domain of 26S rDNA was amplified with primers NL-1 (5′- GCA TAT CAA TAA GCG GAG GAA AAG-3′) and NL4 (5′-GGT CCG TGT TTC AAG ACG G-3′). Amplification was performed in 100 µl of reaction mixture conditioning 100 ng of 2.5 U of Taq polymerase, genomic DNA, 40 mM of each primer, 20 mM of each dNTP, 1.5 mM MgCl2, and 10 mM Tris–HCl. The nucleotide sequences of the 26S rDNA D1/D2 domain were determined directly using PCR products, with slight modifications. Cycle sequencing of the D1/D2 domain was carried out with the forward primer NL1 (5′-GCA TAT CAA TAA GCG GAG GAA AAG-3′) and reverse primer, NL4 (5′-GGT CCG TGT TTC AAG ACG G-3′), using an ABI PrismTM BigDyeTM Terminator Cycle Sequence Ready Reaction Kit (Applied Biosystems, Stafford, USA) according to the manufacturer’s instructions.

### Animals and experimental design

This study used 6 multiparous crossbreds between Holstein Frisian × Zebu dairy cows in their mid-lactation period (165.5 ± 44.0 DIM) with an initial body weight of 363.9 ± 55.80 kg (average milk yield 8.58 kg/day) and a mean age of 5 years. The milk yield reported was slightly higher than the previous studies, in which Holstein Frisian × Zebu cow’s milk yields were 2,897 kg/year or 8.05 kg/day^51^. Dairy cows were randomly allocated to three ensiled RS with various yeast species including *S. cerevisiae*, *P. kudriavzevii* KKU20, and *C. tropicalis* KKU20 according to a 3 × 3 replicated Latin square design.

### Ensiling rice straw with yeast from rumen fluid

**Figure 4:**
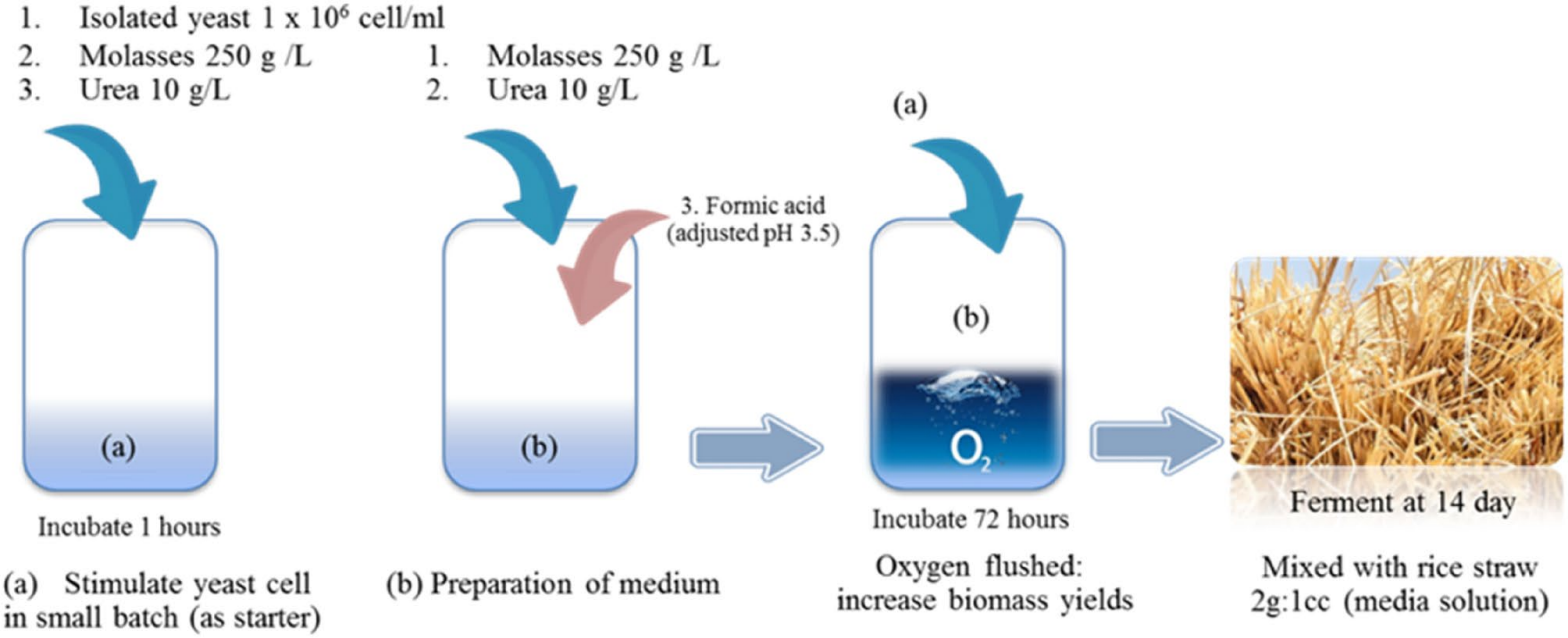
The process of rice straw fermentation with isolated yeast from rumen fluid.

The ruminal yeasts were obtained by isolating, screening, and identifying the rumen of crossbred Thai-Holstein Friesian dairy cattle^6^. The *P. kudriavzevii* KKU20 and *C. tropicalis* KKU20 (considered as Crabtree negative yeast^25^) were tested for their high-potential on in vitro study, which has an outstanding benefit for feed digestion and in vitro gas production^6^. The *S. cerevisiae* (considered as Crabtree positive yeast^25^) was obtained from the commercial baker’s yeast (PERFECT YEAST Co., Ltd, Ubon Ratchathani, Thailand). In Fig. 4, isolated homogenous yeast suspension from the rumen (about 10^6^ cells/mL) was multiplied in media solution including 250 g molasses (Khon Kaen dairy cooperative Co., Ltd., Khon Kaen, Thailand) plus 10 g urea per 1000 mL of water. After that, the solution’s pH was then modified using formic acid (L.C. Industrial Co., Ltd, Nakhon Pathom, Thailand) to reach a final pH of 3.5^52^. Media solution directly into electromagnetic air compressor (HAILEA ACO-318 oxygen pump, Sagar aquarium, Gujarat, India) flushed with oxygen to complete respiration for maximum cell growth at 72 h (final estimated yeast as 1 × 10^9^ CFU/mL^6^. The media solution was mixed on the RS (2:1 ratio) and adjusted with a moisture content of 650–750 g/kg to provide sufficient ensilage conditions^24^. Fifteen kilograms of ensiled RS were put into plastic bags (size 24 × 42 inches, P.P Plastic Pagchong Co., Ltd, Nakhonrachasrima, Thailand), and sealed with a vacuum machine (IMAFLEX 1400 W VC-921, Imarflex Industrial Co., Ltd., Bangkok, Thailand). To ensure an anaerobic environment, the bags were securely sealed and fermented at room temperature for 14 days^6^.

### Feeding and samples collection

The feeding trial lasted for 63 days (21 days/period with 3 periods); dairy cows were held in independent pens and individually fed roughage and concentrate diets at 07:00 and 16:00. Ensiled RS offered ad libitum for all cows. The experimental diet was formulated by using the Khon Kaen Complete Feed (KCF) 2006 Program base on NRC^15^. The ingredients and nutrient composition of ensiled RS and concentrate diet were provided in Table 1. During the experiment, mineral blocks and fresh water were accessible. The experiment was performed over 3 periods with double squares. The period lasted for 21 days, the first 14 days for treatment adjustments and the last 7 days for sample intake and collection assessment.

At the time of the feeding trial, orts were obtained and weights were collected every day, and the feeding rate was adjusted daily to yield orts between 50 to 100 g/kg of intake. Individual voluntary feed determined consumption difference between the feed offered and orts. Around 5 g/kg of the overall fresh fecal samples were split into two parts; the first part of each day for DM analysis and the second part were pooled at the end of each period. The pooled fecal samples (500 g) were stored at − 20 °C until analysis. At 60 °C, composite samples were dried, pressed through a steel filter of 1 mm for grinding (WILEY MILL, Arthur H. Thomas Co., Ltd., Philadelphia, PA, USA), and then analyzed for DM (ID 967.03), ash (ID 492.05), ether extract (EE; ID 455.08), CP (ID 984.13) content^53^, NDF, ADF^54^, and acid-insoluble ash (AIA)^55^. Body weights were measured every period. The calculation of ME was based on the equation defined by Wachirapakorn et al.^32,56^:

1 $${\text{ME}}\;\left( {{\text{MJ}}/{\text{kg}}\;{\text{DM}}} \right) = 0.{82} \times \left[ {{2}.{4} \times {\text{CP}} + {3}.{9} \;{\text{EE}} \times {1}.{8} \times {\text{the}}\;{\text{rest}}\;{\text{of}}\;{\text{the}}\;{\text{OM}}} \right] \times in\;vitro\;{\text{organic}}\;{\text{matter}}\;{\text{digestibility}}\;\left( {{\text{IVOMD}}} \right)$$

where CP, EE, and OM are in g per kg of DM and IVOMD with the mean values received from our recent in vitro study with mean values of 682.5 g/kg DM^6^.

The 10 g of fresh silage was blended with 90 mL of sterilized water and stored at 4 °C^57,58^. The pH of the ensiled RS was measured by a pH meter using cold-water extracts (HANNA HI-8424 Portable pH/ORP Meter, Woonsocket, USA) according to Pholsen et al.^59,60^. Silage fluid subsamples were centrifuged for 15 min at 6,000 rpm and the liquid above the solid residue was filtered using a 0.45-micron syringe filter. High-performance liquid chromatography (HPLC) devices (SHIMADZU LC-20A, Shimadzu Industrial Systems Co., Ltd, Kyoto, Japan) were used to conduct LA, C_2_, C_3_, and C_4_ analyses^61^. The NH_3_-N concentration was calculated according to the Kjeldahl process^53^. Jugular blood and rumen fluid samples were obtained at 0 and 4 h after feeding on the last day of each period. A blood sample (approximately 10 mL) was obtained in tubes containing 12 mg of ethylene diamine tetra-acetic acid (EDTA) from the jugular vein. The plasma was isolated by centrifugation for 10 min at 500×*g* and preserved at − 20 °C until BUN analysis, according to Abdallah et al.^62^. Approximately 200 mL of rumen fluid was collected from the rumen by a stomach tube connected to a vacuum pump. Rumen fluid was assessed immediately by the pH meter for determining the pH and temperature. Rumen fluid samples were then filtered through 4 cheesecloth layers. A fluid sample containing 5 mL of 1 mol/L of H_2_SO_4_ applied to 45 mL of rumen fluid was put into the bottle. The rumen fluid mixture was centrifuged for 15 min at 6000×*g* and used for analyzing the NH_3_-N^53^ and VFA (gas chromatography, MODEL HP6890-HEWLETT, NY, USA)^63^. Ruminal bacteria, protozoa, and fungal zoospores were numbered under a hemocytometer using the direct counting method^64^.

During the last 7 days of each experimental period, milk samples were taken according to the yield for morning and afternoon milking, preserved with 2-bromo-2 nitropropane-1, 3-dial, and stored at 4 °C until analysis by using Milko-Scan (FOSS ELECTRIC, Hillerod, Demark) for fat, true protein, lactose, total solids (TS), and solids-not-fat (SNF) content. Milk urea nitrogen (MUN) was estimated by the diacetyl monoxime method using UV/Vis-spectrophotometer (PG Instruments Ltd., London, UK) according to Aguilar et al.^65^. Fat, protein, lactose, TS, and SNF concentrations were measured as weighted media depending on morning and afternoon milk yields per each test day by infrared methods using Milko-Scan 33.Yields of 4.0% FCM were calculated according to Rafferty et al.^66^:

2 $${\text{FCM}}\; {\text{fat}}\;{\text{ corrected }}\;{\text{milk}} = 0.{432}\;\left( {{\text{kg}}\;{\text{of}}\;{\text{milk}}/{\text{day}}} \right) + {16}.{23}\;\left( {{\text{kg}}\;{\text{of}}\;{\text{fat}}} \right)$$

while yields of ECM were calculated as described by Krause and Combs^67^:

3 $${\text{ECM}}\;{\text{ energy}} - {\text{corrected }}\;{\text{milk}} = {7}.{2}0 \times {\text{protein}}\;\left( {{\text{kg}}/{\text{day}}} \right) + {12}.{95} \times {\text{fat }}\;\left( {{\text{kg}}/{\text{day}}} \right) + 0.{327} \times {\text{milk}}\;\left( {{\text{kg}}/{\text{day}}} \right)$$

For each cow and period, feed conversion efficiencies were determined by dividing the average yield of actual milk and ECM by the respective DMI and digestible protein per yield of actual milk^65^.

### Statistical analysis

All data from the experiment were analyzed according to a 3 × 3 replicated Latin square design using the GLM procedure^68^ according to the model:

$$Yijkl = \mu + Sl + Mi\left( l \right) + Aj + Pk + \varepsilon ijkl$$

where *Yijk*, observation from cow *j*, receiving ensiled RS *i*, in period *k*; *μ*, the overall of mean, *Sl*, the effect of square (*l* = 1, 2); *Mi*, effect of yeast species in RS silage (*i* = 1, 2, 3); *Aj*, the effect of cows (*j* = 1, 2, 3, 4, 5, 6); *Pk*, the effect of period (*k* = 1, 2, 3); and *εijk*, the residual effect. Significant differences between individual means were evaluated using Duncan’s multiple comparison tests when a significant (P < 0.05) effect was detected^69^. Standard errors of means were calculated from the residual mean squares in the analysis of variance.

## Abbreviations

AA: Amino acids
ADF: Acid detergent fiber
ADFD: Acid detergent fiber digestibility
ADL: Acid detergent lignin
AFB1: Aflatoxin type B1
AIA: Acid-insoluble ash
BUN: Blood urea nitrogen
C_2_: Acetic acid
C_3_: Propionic acid
C_4_: Butyric acid
CEL: Cellulose
CP: Crude protein
CPD: Crude protein digestibility
CPI: Crude protein intake
DCP: Digestible crude protein
DIM: Day in milk
DM: Dry matter
DMD: Dry matter digestibility
DMI: Dry matter intake
ECM: Energy corrected milk
EDTA: Ethylene diamine tetra-acetic acid
EE: Ether extract
FCM: Fat corrected milk
GE: Gross energy
HCEL: Hemicelluose
HPLC: High-performance liquid chromatography
IVOMD: In vitro organic matter digestibility
KCF: Khon Kaen complete feed
KKU: Khon Kaen University
LA: Lactic acid
ME: Metabolizable energy
MPC: Microbial crude protein
MUN: Milk urea nitrogen
NDF: Neutral detergent fiber
NDFD: Neutral detergent fiber digestibility
NH_3_-N: Ammonia nitrogen
NRC: National Research Council
OM: Organic matter
OMD: Organic matter digestibility
PDH: Pyruvate dehydrogenase
RS: Rice straw
SCC: Somatic cell count
SNF: Solids-not-fat
TDN: Total digestible nutrient
TS: Total solids
TVFA: Total volatile fatty acid
VFA: Volatile fatty acid
